# Supplementary material for: Identifying potential biomarkers and therapeutic targets for dogs with sepsis using metabolomics and lipidomics analyses
Source: PLoS One. 2022 Jul 8;17(7):e0271137. doi: 10.1371/journal.pone.0271137 (PMC9269464; doi:10.1371/journal.pone.0271137)
Supplement: S1 Data — (PDF) [file pone.0271137.s001.pdf]

IS Standards used to make ISTD	Vendor	Working concentration in DCM/METOH (2:1 v/v)
TG $(15:0)_3$	Sigma T4257	25 µg/mL (0.0326 mM/L)
PG $(14:0)_2$	Avanti 840445-01-167	25 µg/mL (0.0362 mM/L)
PS $(16:0)_2$	Avanti 840037-01-076	25 µg/mL (0.0109 mM/L)
Cer (d18:1_12:0)	Avanti 860512P-10mg-A-010	25 µg/mL (0.0518 mM/L)
ChE (17:0)	Avanti 700186M-1mg-F-011	25 µg/mL (0.0195 mM/L)
LysoPC (18:1D7)	Avanti 791643C-1mg-d-010	5 µg/mL (0.0236 mM/L)
PC (18:1D7_15:0)	Avanti 791637C-1mg-d-010	5 µg/mL (0.0165 mM/L)
