# Supplementary figures and images for: Identifying potential biomarkers and therapeutic targets for dogs with sepsis using metabolomics and lipidomics analyses
Source: PLoS One. 2022 Jul 8;17(7):e0271137. doi: 10.1371/journal.pone.0271137 (PMC9269464; doi:10.1371/journal.pone.0271137)

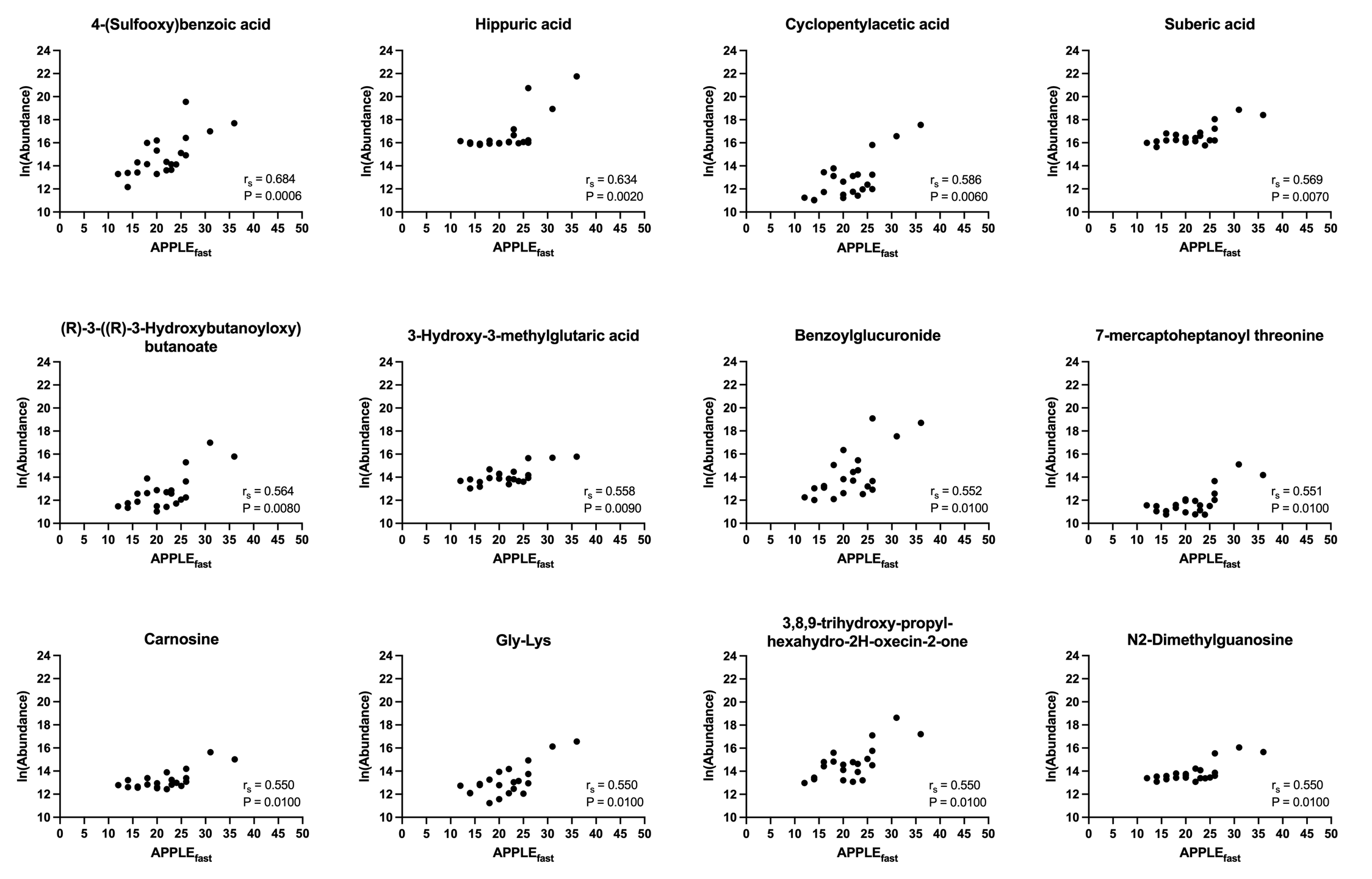

Supplement: S1 Fig — Scatterplots were constructed to explore the relationships between illness severity as assessed by the APPLEfast score and prognostic biomarker abundance. Displayed are the 12 biomarkers with the highest degree of correlation with the APPLEfast score. The associated Spearman correlation coefficient (rs) and corresponding unadjusted P-value is displayed inset on each scatterplot. (TIF) [file pone.0271137.s004.tif]
