# Supplementary material for: Identifying potential biomarkers and therapeutic targets for dogs with sepsis using metabolomics and lipidomics analyses
Source: PLoS One. 2022 Jul 8;17(7):e0271137. doi: 10.1371/journal.pone.0271137 (PMC9269464; doi:10.1371/journal.pone.0271137)
Supplement: Attachment — Submitted filename: Identifying potential biomarkers and therapeutic targets for dogs with sepsis by parallel metabolomics and lipidomics analyses_PlosOne_April2022.docx [file pone.0271137.s005.docx]

The manuscript “Identifying potential biomarkers and therapeutic targets for dogs with sepsis by parallel metabolomics and lipidomics analyses” reports metabolic signatures of sepsis in dogs. The dataset generated from this study will contribute to the canine metabolome database and provide knowledge on septic biomarkers. However, variability within the study populations due to several factors and lack of information on control groups make it unsuitable to publish in its current form. If the authors can provide relevant information and clarify below comments it can be considered for publication in PLosOne.

Major comments:

1. The authors have not mentioned anything about the breed of the dogs used in this study. Given that breed variability in dog plasma metabolome has been reported, this is important to understand how it impacts the data. In addition, there is no information on the breed, age, gender information of the control group.
2. Line 267, Table 1 is confusing with the same variables represented by two different units (SI and US). Is there any specific reasons for showing the same data in two formats? It will more informative to provide comparative values from the control group along with the sepsis group with either US or SI units (if needed the other unit can be given as supplementary data).
3. The title reads “….by parallel metabolomics and lipidomics analyses”. However, the authors have used separate sample extraction methods for small molecule metabolomics and lipidomics instead of using a bi-phasic extraction and subjecting respective phases to metabolomics and lipidomics parallelly. Why? please comment and change title.
4. In the methods section, internal standards were added (after extraction) during resuspension of dried extract for untargeted metabolomics while for untargeted lipidomics internal standards were added during the extraction step. As the latter is the recommended method, why this difference in sample preparation?
5. The authors have acknowledged the diverse diets of sepsis dogs used in this study but have not mentioned the diet of control dogs. As diet influences the metabolome significantly, to make sense of the comparison with control group, this information and any correlation to it is important. Please report information of control group diet. Also, wondering if there was a fasting time (for a short period) for animals prior to sample collection.
6. It will be interesting to check for any correlations between the identified putative metabolic biomarkers and sepsis causes/severity.
7. This study discusses dog sepsis metabolome data in context with human sepsis metabolome data. It will add value to include a venn diagram of metabolic biomarkers identified in this study and those from human sepsis metabolome studies. It will give a visual perspective of unique and common metabolic biomarkers identified in this study.

Minor comments:

1. Line 276: OPLS-DA is mentioned in the test while PCA is mentioned in the figure legend 2 A. Please clarify. Assignment of figure number is not similar in the text (2A is OPLS-DA) and figure legend (2A is volcano plot).
2. Please provide high resolution images.
